# A Unique Resource Mutualism between the Giant Bornean Pitcher Plant, *Nepenthes rajah*, and Members of a Small Mammal Community

**DOI:** 10.1371/journal.pone.0021114

**Published:** 2011-06-14

**Authors:** Melinda Greenwood, Charles Clarke, Ch'ien C. Lee, Ansou Gunsalam, Rohan H. Clarke

**Affiliations:** 1 School of Biological Sciences, Monash University, Clayton, Victoria, Australia; 2 School of Science, Monash University, Bandar Sunway, Selangor, Malaysia; 3 Kuching, Sarawak, Malaysia; 4 Sabah Parks, Kota Kinabalu, Sabah, Malaysia; Texas A&M University, United States of America

## Abstract

The carnivorous pitcher plant genus *Nepenthes* grows in nutrient-deficient substrates and produce jug-shaped leaf organs (pitchers) that trap arthropods as a source of N and P. A number of Bornean *Nepenthes* demonstrate novel nutrient acquisition strategies. Notably, three giant montane species are engaged in a mutualistic association with the mountain treeshrew, *Tupaia montana*, in which the treeshrew defecates into the pitchers while visiting them to feed on nectar secretions on the pitchers' lids.

Although the basis of this resource mutualism has been elucidated, many aspects are yet to be investigated. We sought to provide insights into the value of the mutualism to each participant. During initial observations we discovered that the summit rat, *R. baluensis,* also feeds on sugary exudates of *N. rajah* pitchers and defecates into them, and that this behavior appears to be habitual. The scope of the study was therefore expanded to assess to what degree *N. rajah* interacts with the small mammal community.

We found that both *T. montana* and *R. baluensis* are engaged in a mutualistic interaction with *N. rajah*. *T .montana* visit pitchers more frequently than *R. baluensis*, but daily scat deposition rates within pitchers do not differ, suggesting that the mutualistic relationships are of a similar strength. This study is the first to demonstrate that a mutualism exists between a carnivorous plant species and multiple members of a small mammal community. Further, the newly discovered mutualism between *R. baluensis* and *N. rajah* represents only the second ever example of a multidirectional resource-based mutualism between a mammal and a carnivorous plant.

## Introduction

The carnivorous pitcher plant genus *Nepenthes* (Nepenthaceae) consists of ∼120 species that are predominantly found in the Southeast Asian tropics [1], [2]. Most species grow in nutrient-deficient substrates and produce jug-shaped leaf organs that trap arthropods as a supplementary source of N and P which are otherwise scarce in their habitats [3]. Diversity in *Nepenthes* – in terms of both species numbers and trap form – is greatest in Borneo, where some of the largest and most unusual species occur [4].

Recently, it has been shown that several Bornean *Nepenthes* species demonstrate specialized nutrient acquisition strategies, which differ markedly from the ‘typical’, arthropod-trapping strategy [5], [6]. Clarke *et al.* [7] and Chin *et al.* [8] established that three giant montane pitcher plant species from Borneo, *Nepenthes lowii* Hook.f., *N. rajah* Hook.f. and *N. macrophylla* (Marabini) Jebb & Cheek, are engaged in an extraordinary mutualistic association with mountain treeshrews (*Tupaia montana* Thomas (Scandentia)), in which the treeshrews defecate into the *Nepenthes*' pitchers while visiting them to feed on carbohydrate rich secretions produced by glands on the pitchers' lids.

Although the basis of this mutualism has been elucidated, many aspects have yet to be investigated [6]. For instance, the behavioral ecology of *T. montana* with respect to *Nepenthes* has not been studied in detail. No observation of pitcher visitors or nectar production by pitchers has been attempted at night. We do not know whether individual treeshrews defend valuable pitchers against other animals, or whether such resources are shared. There is almost no quantitative data relating to the frequency, duration and interval between visits by *T. montana*, or the rate of scat deposition into *Nepenthes* pitchers.

In this study, we conducted a series of experiments and observations designed to provide further insights into the interaction between *T. montana* and *N. rajah* on Mount Kinabalu, Sabah, Malaysian Borneo. Our objective was to determine the time of day, frequency, duration, and interval between pitcher visits by *T. montana*, along with rates of scat deposition and preliminary measures of diurnal and nocturnal nectar sugar content, to provide the necessary foundations for future experiments designed to quantify the value of the mutualism to each participant. However, during our preliminary observations, we detected a second mammalian visitor to *N. rajah* pitchers, the summit rat (*Rattus baluensis* Thomas (Rodentia)). We discovered that like *T. montana*, *R. baluensis* feeds on the lid gland exudates of *N. rajah* pitchers and defecates into them, and that this behavior appears to be habitual. This raised the possibility that *N. rajah* interacts with a community of small mammal species, so we expanded the scope of our study, comparing the visiting behavior of *T. montana* with that of *R. baluensis* at *N. rajah* pitchers.

Specifically, we investigated what time of day (or night) *T. montana* and *R. baluensis* visited *N. rajah* pitchers and whether there was any evidence for competition or avoidance between mammalian visitors. The visitation rate, duration of visits and interval between visits was also investigated to explore similarities and differences between the two species. Finally, this study sought to document the scat deposition rates within pitchers to explore the strength of the mutualistic relationship between each mammal species and *N. rajah*.

We found that both *T. montana* and *R. baluensis* are engaged in a mutualistic interaction with *N. rajah*, that *T. montana* visits pitchers more frequently than *R. baluensis*, but that daily scat deposition rates within pitchers do not differ between the two species. This study is the first to demonstrate that a mutualism exists between a single species of carnivorous plant and multiple species of small facultative mammalian nectarivores.

## Materials and Methods

### Ethics statement

As this was an observational study of free ranging wild animals, with no direct interaction between the observers and animals, ethics clearance was not required. The work was conducted in Sabah, Malaysia in accordance with an Economic Planning Unit Permit (0/200/19/2545) and a Sabah Parks Research Permit (TS/PTD/5/4 Jld/ 39 (37)) held by the authors. We thank Dr Maklarin Lakim, Rimi Repin and Sabah Parks for assistance and permission to conduct the research.

### Study site

All research was conducted at the ‘Mesilau Landslip’ on Mount Kinabalu, Sabah, Malaysia (6.048°N, 116.599°E, 2050 m asl). The study site is approximately 70×50 m in size and is located on an east-facing slope on the land slip, over an ultramafic substrate, with patchy and stunted vegetation, surrounded by lower montane forest. The sparse over-story is coniferous and *N. rajah* plants grow among sedges, ferns and small shrubs.

### Selection of pitchers for use in experiments

All plants of *N. rajah* that were readily accessible and located outside patches of fragile vegetation were tagged and their pitchers examined at regular intervals throughout the study period. Very young pitchers that have just opened have soft tissues and do not appear to be visited frequently by vertebrates [7], [8] so these were excluded. Based on our observations, and those of Chin *et al.* [8], *N. rajah* pitchers appear to have a functional lifespan of three to six months, with the majority of vertebrate visits occurring over the first three months. Older pitchers frequently exhibit structural damage, partial necrosis, and extensive fungal growth on regions where nectar is secreted, and these signs were used to exclude older pitchers from experiments, based on the assumption that older pitchers are visited by vertebrates infrequently and inconsistently [8]. As a single plant can support multiple pitchers, within any given experiment, all pitchers used were on separate plants.

### Filming of pitcher visitors

Filming of pitchers was conducted between the 18 July and the 16 November 2010. Combinations of video cameras were used to film one to two *N. rajah* pitchers on each day, with filming during daytime starting at sunrise and ending at sunset whenever possible. However, during periods of adverse weather or equipment failure, this schedule could not always be adhered to. Filming during nighttime was limited by the availability and battery life of video cameras with night-recording capabilities and logistical constraints.

Visits by vertebrates to *N. rajah* pitchers were filmed using digital video cameras and a still camera trap. Several digital video cameras were used: Sony HDR-XR550 with Infrared light (HVL-HIRL), Sony HDR-CX150, Sony DCR-SX44, Sony Corporation, Tokyo, Japan and Panasonic SDR-S7, Panasonic Corporation, Osaka, Japan) mounted on tripods.

Filming methods described elsewhere [7], [8] were replicated with increased effort during daylight hours. Unlike previous work, filming also included nocturnal sampling periods. Total filming effort was 515.1 hours between sunrise and sunset, and 44.6 hours between sunset and sunrise. Sunrise occurred between 6:01–6:09am, and sunset between 5:54–6:33pm (Malaysia Time) during the filming period [9]. Sixty-nine individual pitchers were filmed over the course of the study.

Between 4 and 6 August, 2010 camera trapping was conducted during the hours of darkness at two pitchers such that a total of 48 hours of nocturnal monitoring was achieved. Each camera was positioned on a tripod approximately 1.5 m from the pitcher (Nikon D90 D-SLR, Nikon Corporation, Tokyo, Japan), with the camera trap beam set to maximum sensitivity and crossing the pitcher mouth just above the peristome (Phototrap photographic trigger system model 33, Phototrap, Amado, Arizona), Nikon SB-900 off-camera flash).

### Measurement of scat inputs to pitchers

Scat input to pitchers was monitored by mounting a thin plastic barrier (hereafter referred to as a ‘cup’) across the orifice of the pitcher. The barrier was positioned below the level of the peristome (a collar-like ridge of hardened tissue that lines the pitcher orifice [4]) a few millimeters above the surface of the fluid within the pitcher and was held in place with small magnets. Scats deposited into the pitcher were trapped in the cups, and were counted and removed daily for 38 days between the 26 September and the 17 November, 2010. Initially, the origin of the scats was determined using video recordings, but as we found that the scats of *T. montana* and *R. baluensis* differed consistently in size, colour, consistency and odour subsequent determinations were made visually (n = 20). *T. montana* scats were dark brown to black, consisted of heterogeneous components that were relatively friable and had a strong unpleasant odour, whereas *R. baluensis* scats were pale brown, with the contents appearing homogenous, relatively firm and with a mild odour.

### Preliminary measurements of pitcher nectar sugar concentration

Nectar produced by glands on the pitcher lids was sampled during both day and night, to determine the gross sugar concentration of the nectar. The lids of pitchers used in these observations were initially cleaned using a damp tissue, and then enclosed within a mosquito net. Using gloved hands, pitcher lids were swabbed with half a single ply tissue, and captured nectar was transferred to a 2 ml eppendorf tube. Any remaining nectar was wiped off the pitcher lid using a clean tissue before repositioning and securing the netting. The gross sugar concentration of individual nectar samples was measured (in Brix) using a digital hand-held refractometer (Atago, PAL-1 0–53% Pocket Refractometer, Atago Corporation, Tokyo, Japan) (n = 18).

### Statistical analysis

Data were tested for normality (Shapiro Wilk Normality test) and boxplots were constructed and scrutinized to ensure homoscedasticity. Where necessary, data were transformed to meet these assumptions. Variables that failed to meet these assumptions when transformed were analyzed using non-parametric methods. Decisions about hypotheses were made against a statistical criterion of α = 0.05. Analyses were conducted using Minitab v12.23.

## Results

### *Nepenthes rajah* pitchers are routinely visited by two species of mammal

**Figure 1:**
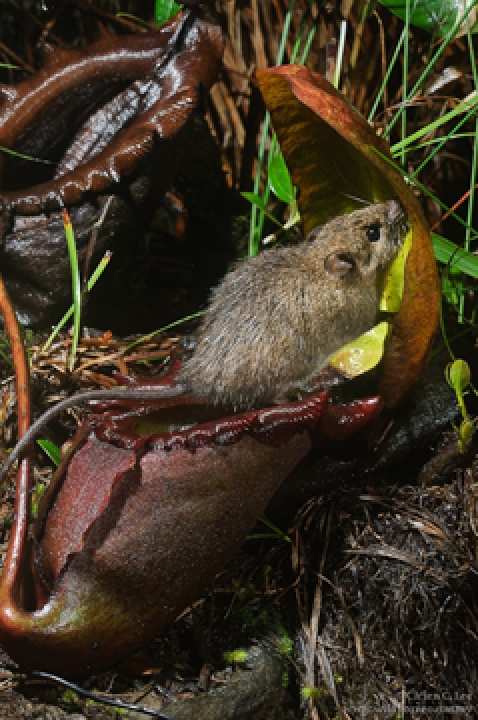
*Rattus baluensis* visiting a *Nepenthes rajah* pitcher at night.

We found that in addition to *T. montana*, the summit rat, *Rattus baluensis*, habitually visits *N. rajah* pitchers to feed on nectar produced by glands on the pitcher lids (Fig. 1). Like *T. montana*, *R. baluensis* frequently deposits scats into *N. rajah* pitchers. Despite an intensive monitoring effort, no other vertebrates were observed to exploit nectar resources nor defecate in pitchers during this study.

### Visitation to pitchers by a small mammal community

Using video, a total of 238 *T. montana*, and 18 *R. baluensis* visits were recorded visiting pitchers. Using camera traps nine *R. baluensis* were detected visiting pitchers. A single nocturnal recording of a *R. baluensis* visit that lasted for 18.27 minutes was removed from all duration analyses as the animal displayed atypical behavior not normally associated with feeding and defecating.

### Timing of visitation to pitchers

**Figure 2:**
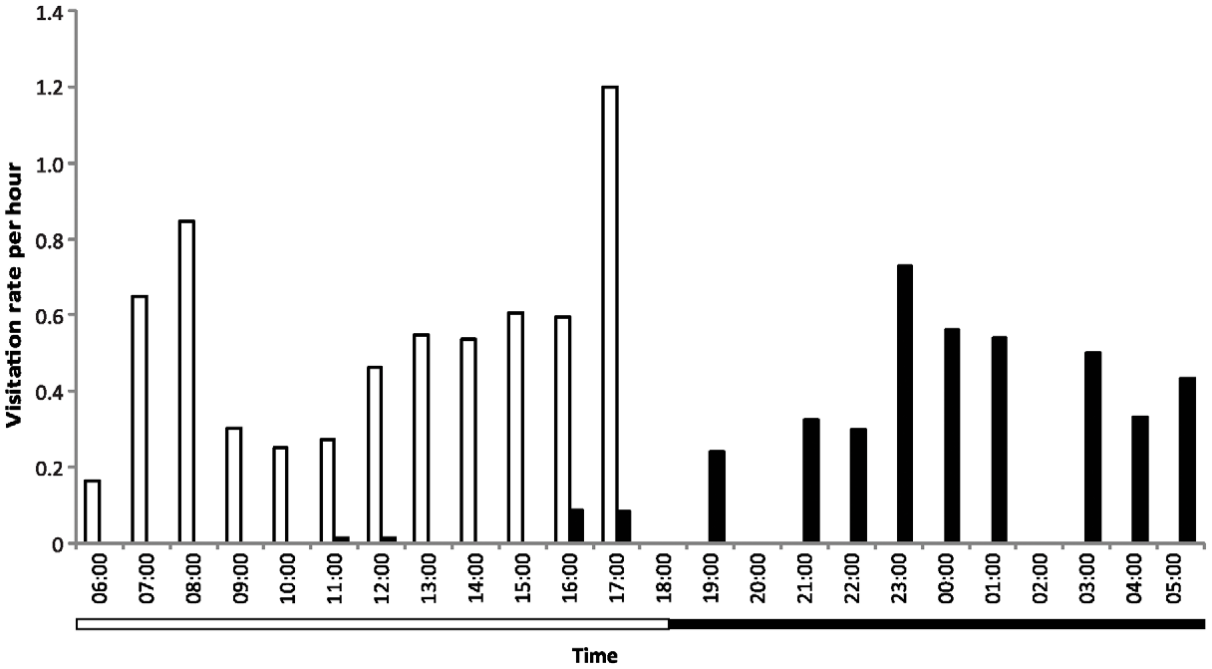
Small mammal visitation rates to pitchers by hour of observation over the complete diel cycle. Open bars: *T. montana*, closed bars: *R. baluensis*. The continuous bar under the x-axis represents photoperiod - white: light, black: dark.

All *T. montana* visits (n = 238) occurred during daylight hours, indicating that *T. montana* is strictly diurnal. In contrast, the 27 *R. baluensis* visits occurred during both daylight (six visits) and at night (21 visits), showing that although this species appears to be largely nocturnal, it is on occasions active at various times during the diel period (Fig. 2).

### Rate of visitation to pitchers

During daylight hours, *T. montana* visited pitchers at a rate of 0.46 visits per hour, whereas *R. baluensis* visited at a rate of 0.01 visits per hour. At night the visitation rate for *R. baluensis* was 0.31 visits per hour. There was no significant difference in median visitation rate per hour between *R. baluensis* and *T. montana* (Paired samples Wilcoxon test, W = 266.0, df = 47, p = 0.064) (Fig. 2). The mean daily (24 hour) rate of visitation for *T. montana* was 5.54 visits per day whilst for *R. baluensis* it was 3.81 visits.

### Duration of time spent on pitchers

For data involving all recorded visits, there was no significant difference between the mean duration that *T. montana* remained at pitchers (mean  = 19.91±16.14 s, n = 238) compared to that of *R. baluensis* (mean  = 35.18±42.23 s, n = 17) (student t-test conducted on log-transformed data, t = −1.1844, df = 253, p = 0.237). Linear regression by ordinary least squares demonstrated that there was no significant relationship between time after sunrise and duration of visit. The mean duration of visits by both mammal species to *N. rajah* pitchers was not related to time of day (OLS regression on log-transformed data, F_1,20_ = 0.676, p = 0.421).

On several occasions, more than one *T. montana* visit was detected on a single continuous video recording. Sixty-six recordings detected two visits, 24 recordings detected three visits and nine recordings detected four or more visits. The mean durations of visits 1, 2 and 3 were 24.67±16.44 s, 19.00±15.78 s and 18.29±13.75 s, respectively. There were no significant differences among these means (ANOVA F_2,69_ = 1.310, p = 0.276). The difference in the mean interval between first and second (mean for interval one  = 54.82±41.38 min) or second and third (mean for interval two  = 49.45±35.70 min) visits was also non significant (student t-test, t = 0.941, df = 86, p = 0.350).

### Differences in location of scat deposition, by species

**Figure 3:**
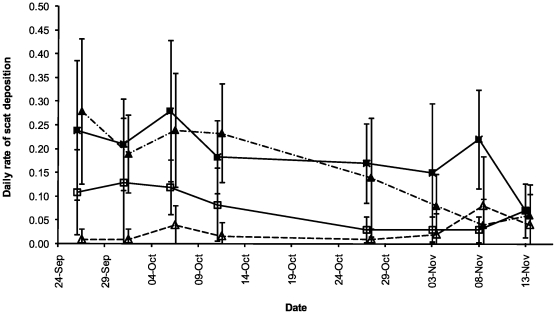
Mean rate of scat deposition to pitchers by *Tupaia montana* and *Rattus baleunsis*. Closed squares: Mean *T. montana* scats inside pitchers, open squares: mean *T. montana* outside pitchers, closed diamonds: mean *R. baluensis* scats inside pitchers, open diamonds: mean *R. baluensis* scats outside pitchers.

In pitchers that contained plastic cups, *T. montana* deposited 145 scats inside and 57 scats outside pitchers, whereas *R. baluensis* deposited 117 scats inside and 22 scats outside pitchers. The pattern of deposition of scats (inside versus outside) was significantly different between the two mammal species (χ^2^ = 6.48, df = 1, p = 0.008), with *R. baluensis* demonstrating a greater degree of ‘accuracy’, depositing only 16% of scats outside the pitchers (c.f. 28% for *T. montana*). This pattern is reflected in the daily rates of scat deposition (both into and adjacent to pitchers), which varied little throughout the study period (Fig. 3).

### Sugar concentrations of nectar produced by the pitcher-lid glands of *N. rajah*

The mean gross sugar concentration of the nectar produced by the lid glands of *N. rajah* during daylight hours was 6.97±1.71% (n = 76). The mean gross sugar concentration of nectar samples collected at night was 7.32±2.84% (n = 76). There was no significant difference in gross sugar concentrations between nectar sampled during the day and at night (Wilcoxon signed-rank test, T+ = 1179.5, T− = 1517.5, T_0.05(2)_,71 = 936, n = 73, P = >0.050).

## Discussion

Our findings demonstrate that a mutualism exists between a species of carnivorous plant and *multiple* facultative mammalian nectarivores. That multiple members of the small mammal community present at this site are involved in a resource-based mutualism is an exciting discovery. Further, the mutualism between *R. baluensis* and *N. rajah* represents only the second known example of a multidirectional resource-based mutualism between a mammal and a carnivorous plant, and the third documented instance of a mutualism of any sort between *Nepenthes* and mammals [7], [10].

This study has also identified *N. rajah* as the first *Nepenthes* species that appears to benefit from mammalian fecal inputs to its pitchers during both daylight hours and at night. This likely represents a significant nutritional benefit to *N. rajah* that is not available to its congeners, *N. lowii* and *N. macrophylla*, as the latter species appear to receive fecal inputs only from the diurnal *T. montana* [8]. This contention is further supported by the fact that *R. baluensis* is a range restricted Mount Kinabalu endemic [11] and as such its distribution does not overlap with that of *N. lowii* and *N. macrophylla*. As pitcher visitation and scat deposition rates in *N. rajah* pitchers by the diurnal *T. montana* and the nocturnal *R. baluensis* are approximately equal, the rate of supplementary nutritional inputs to *N. rajah* pitchers is potentially much higher than in other *Nepenthes* species. In a similar mutualism between *N. lowii* and *T. montana* it has been estimated that between 57 and 100 per cent of foliar N in *N. lowii* plants is derived from feces [7]. This raises the possibility that foliar N studies involving *N. rajah* may demonstrate even higher minimum contributions to foliar N by the small mammal community at Mount Kinabalu.

Segregation of visiting times between *T. montana* and *R. baluensis* may confer benefits to all three active participants in the mutualism. The lid glands of *N. rajah* produce quantities of dilute nectar during both the day and night. Regular consumption of this resource reduces potential nectar loss from the pitcher lid via other avenues (e.g. run off). This in turn maximizes potential benefits to the plant by ensuring almost all nectar that is produced is available to mutualists capable of depositing N rich fecal matter within pitchers. As *T. montana* has been shown to consume all secreted nectar on the lids of *N. rajah* pitchers during a single visit [6], [7], competition for pitcher nectar among mammals is potentially intense. As such, temporal segregation of pitcher visits by the two mammal species enables *T. montana* and *R. baluensis* to exploit the same resource whilst largely avoiding direct conflict.

The mean sugar concentrations of nectar exuded during both daylight hours and at night were similar. As a consequence, at this preliminary stage, the relationship between each mammal species and *N. rajah* appears to be equally balanced despite being strongly segregated by time of day. Recognizing that hexoses (e.g. fructose and glucose) and sucrose components of nectar may vary with regards to the taxon of mutualist utilizing this resource [12], [13], further work to quantify the concentrations of these components would provide more robust insight concerning the observed relationships. *T. montana* appears to be strictly diurnal [14], (this study). In contrast, *R. baluensis* visits pitchers during both the day and night, but the daytime visitation rate (1 visit per 100 hours during daylight c.f.1 visit every 3 hours at night) suggests that it is either much less active during at this time, or that it may be prevented from accessing pitchers more regularly at these times.

*Tupaia montana* normally consume a predominantly arthropod diet, substituted with some fruit [14]. In keeping with this diet the species displays a simple intestinal morphology, resulting in a relatively short digestive tract and a gut passage time loosely correlated with body size – amongst all treeshrews (Tupaiids) gut passage is no longer than 1 hour [14]. One consequence of rapid passage of food particles through the gut is that fewer nutrients are extracted from the diet [14] and more are excreted in the scats. Such rapid gut passage likely results in a particularly nutrient rich fertilizer. Although little is known about the gross anatomy of *R. baluensis*, preliminary analysis of fecal matter suggests that they too are omnivorous at our study site (M. Greenwood unpubl. data). In addition to generalized omnivory, *T. montana* has been reported to consume large quantities of wild fruits and berries from which sugar laden juices are extracted [14]. Emmons [14] observed that treeshrews may station themselves at a fruiting plant and feed in short bursts, gorging themselves in order to satisfy the dietary deficiencies of an otherwise arthropod diet. At Mount Kinabalu, fruit gorging behavior by *T. montana* appears to be rare with no direct observations during extended field work (M. Greenwood unpubl. data). An alternative hypothesis that may apply to this population is that nectar feeding *T. montana* obtain many of their carbohydrate requirements from nectar [6], substituting the need for large quantities of fruit that are apparently consumed in other populations [14]. Support for this hypothesis may be found in dietary analysis of both small mammals, with the prediction that reduced fruit consumption would occur in areas where a mutualism with *N. rajah* existed when compared with diet in other areas where *T. montana* and *R. baluensis* persist in the absence of *N. rajah*.

Despite a diverse small mammal fauna in the immediate vicinity of the *N. rajah* population at the Mesilau Landslip (M. Greenwood unpubl. data), just two species of mammal maintain readily detected mutualisms with this species of *Nepenthes*. It is possible that these two species actively monopolize the resource during their preferred diel periods of activity to the exclusion of other potential competitors. If so, this study provides an example of resource partitioning likely facilitated by ancestral behavioral traits (diurnal vs nocturnal activity patterns). Regular visits by the same (possibly patrolling) individuals and observations of scent marking by *T. montana* [7], (this study), provide some insight into the strategies that these two species may employ to protect the resource from competitors that share similar patterns of daily activity but further work in this area is needed.
