# Correction: A Unique Resource Mutualism between the Giant Bornean Pitcher Plant, *Nepenthes rajah*, and Members of a Small Mammal Community

**DOI:** 10.1371/annotation/1ee8d7d0-eff5-431f-9059-c7dbf1bde347

**Published:** 2012-01-03

**Authors:** Melinda Greenwood, Charles Clarke, Ch'ien C. Lee, Ansou Gunsalam, Rohan H. Clarke

Figure 1 has been removed and was replaced with a lower resolution image. The image was taken by author Ch'ien C. Lee and used with his permission.

